# High prevalence of vertebral fractures at initiation of androgen deprivation therapy for prostate cancer

**DOI:** 10.1016/j.jbo.2022.100465

**Published:** 2022-12-07

**Authors:** Marsha M. van Oostwaard, Joop P. van den Bergh, Yes van de Wouw, Maryska Janssen-Heijnen, Marc de Jong, Caroline E. Wyers

**Affiliations:** a Department of Internal Medicine, VieCuri Medical Centre, P.O. Box 1926, 5900 BX Venlo, the Netherlands; b Department of Internal Medicine, NUTRIM School of Nutrition and Translational Research in Metabolism, Maastricht University Medical Centre+ (Maastricht UMC+), P.O. Box 616, 6200 MD Maastricht, the Netherlands; c Department of Urology, VieCuri Medical Centre, P.O. Box 1926, 5900 BX Venlo, the Netherlands; d Department of Clinical Epidemiology, VieCuri Medical Center, Venlo, the Netherlands; e Department of Epidemiology, GROW School for Oncology and Reproduction, Faculty of Health, Medicine and Life Sciences, Maastricht University, Maastricht, the Netherlands

**Keywords:** Prostate cancer, Androgen deprivation therapy, Osteoporosis, Sarcopenia, Vertebral fractures, Fracture risk, PCa, Prostate cancer, ADT, Androgen deprivation therapy, VF, Vertebral fracture, BMD, Bone mineral density

## Abstract

**Purpose:**

Treatment of Prostate Cancer (PCa) with Androgen Deprivation Therapy (ADT) involves long-term consequences including bone loss and fractures. Our aim was to evaluate the calculated fracture risk and the prevalence of osteoporosis, vertebral fractures (VF) and sarcopenia in men with PCa at initiation of ADT, as ADT will increase fracture risk from that moment onward.

**Methods:**

In this cross-sectional real-world study in men at ADT initiation, fracture risk factors including comorbidities, medication, and 10-year fracture risk (FRAX®) were assessed. Laboratory tests, dual-energy X-ray absorptiometry, and spinal X-rays were performed. Sarcopenia was defined according to EWGSOP2.

**Results:**

In 115 men at ADT initiation, aged 73.3 (±7.6) years, osteoporosis was diagnosed in 4.3 % and osteopenia in 35.7 %. The mean 10-year fracture risk of major osteoporotic fracture was 4.4 % and of hip fracture 1.7 %, respectively. At least one VF was present in 32.2 % and 33.9 % of men had osteoporosis and/or a VF assessed on spinal X-rays. In 10.4 % at least one new fracture-risk-associated metabolic bone disorder was diagnosed with laboratory testing. Sarcopenia was diagnosed in only one patient.

**Conclusions:**

Although the prevalence of osteoporosis, sarcopenia and 10-years fracture risk is low, there is a high prevalence of vertebral fractures in a third of the men with PCa at the time of ADT initiation. Besides a BMD measurement and fracture risk calculation using FRAX, a systematic vertebral fracture assessment should be considered in all men with PCa at initiation of ADT to provide a reliable baseline classification of VFs to improve identification of true incident VFs during ADT.

## Highlights

• Low prevalence of osteoporosis, sarcopenia and 10-year fracture risk at ADT initiation.
• High prevalence of vertebral fractures in men with PCa at ADT initiation.
• From the moment of ADT initiation fracture risk will increase.
• Awareness of baseline VF helps identification of new VFs during ADT.
• Assessment of vertebral fractures should be considered at time of ADT initiation.

## 1 Introduction

Worldwide, prostate cancer (PCa) is the most frequently diagnosed type of cancer in males. In the Netherlands, approximately 11,000 patients are diagnosed with PCa every year [1]. Therapies like Androgen Deprivation Therapy (ADT) are prescribed in one in two patients with PCa as curative neoadjuvant therapy combined with radiation therapy, adjuvant therapy following radiation, or continuously in metastatic disease [2]. Although ADT can have significant benefits on survival, the evidence that ADT increases long term fracture risk is convincing [3], [4], [5], [6]. ADT rapidly induces hypogonadism in only 30 to 90 days after the first administration. This sudden decrease of testosterone levels lead to symptoms such as fatigue, weight gain, loss of skeletal muscle-mass and muscle weakness, and bone loss [2], [7].

Bone loss, reflected by a decrease of the bone mineral density (BMD) is most pronounced in the first year after initiation of ADT [7], suggesting that preventive measures to reduce the risk of fractures could be of immediate importance at the time of initiation of ADT. Additionally, ADT is also associated with impaired physical functioning by an impaired muscle mass and strength decreasing directly from the start of ADT, increasing the risk for developing sarcopenia [8], [9], [10] which is associated with falls and fractures in older adults [11]. However, literature on the prevalence of fracture risk factors such as osteoporosis, clinical risk factors sarcopenia and vertebral fractures in PCa patients at the moment of ADT initiation is scarce.

Based on the current guidelines by the European Society for Medical Oncology (ESMO) [12], European Association of Urology (EAU) [13], and a position statement and algorithm by the International Osteoporosis Foundation (IOF) [14], fracture risk assessment in men with PCa ‘starting or receiving’ ADT treatment is recommended, with no specific recommendations on the exact timing of the risk assessment. Overall, in current guidelines fracture risk is considered ‘high’ based on the intervention criteria: BMD and calculated *T*-score and FRAX score (using the FRAX® tool) reaching intervention thresholds [14]. The IOF algorithm also includes prevalent fragility fractures (including vertebral fractures) as an intervention criteria, independent of *T*-score values. Conversely, the presence of prevalent vertebral fractures (VFs) is often not taken into account making it difficult to identify a VF as a ‘new incident’ fragility fracture during ADT treatment course [15]. Despite these quality guidelines, fracture prevention in PCa patients starting or receiving ADT is suboptimal [16], [17].

In this study, reflecting real-life clinical practice, we investigated the calculated fracture risk (FRAX®), the prevalence of osteoporosis, vertebral fractures, and sarcopenia in men with PCa at the time of ADT initiation.

## 2 Patients and methods

### 2.1 Care-pathway

Since January 2019, a multidisciplinary care-pathway involving both experts in bone health and urologists, was initiated in our hospital to assess bone health in men with PCa starting ADT treatment. Patients with known bone metastasis were not part of the care-pathway because of the difference in the preventive therapeutic treatment and treatment goals in patients with bone metastasis (preventing skeletal related events such as pathological fractures and spinal cord compression) or without bone metastasis (bone loss and fragility fractures). The aim of the care-pathway was to assess fracture risk directly at the time of ADT treatment initiation, including assessment of osteoporosis, vertebral fractures, fracture risk related comorbidities and medication, fall risk, and sarcopenia. Based on the outcome of the assessment, lifestyle changes were encouraged, education was provided regarding fracture risk. Treatment with anti-osteoporosis medication (AOM) was initiated when fracture risk was considered high based on the intervention threshold *T*-score ≤ -2.0 and/or ≥ 1 VF grade 2, 3 in accordance with the Dutch guideline on osteoporosis and fracture prevention (https://www.richtlijnendatabase.nl).

### 2.2 Study design

In this cross-sectional study, reflecting real-life clinical practice, men with localised or advanced PCa were included of whom fracture risk was assessed at initiation of ADT in our multidisciplinary care-pathway from January 2019 until December 2020.

Patients previously treated with ADT were excluded and patients with known metastases (M1b) were not included due to the nature of our care-pathway. Local disease was defined as either intermediate risk neoadjuvant ADT prior to radiotherapy, or high risk adjuvant ADT treatment and radiotherapy [13]. Advanced PCa was defined as long-term palliative ADT treatment due to non-skeletal metastasis or biochemical recurrence. Data were extracted from the electronic health records and included date of PCa diagnosis (histological confirmation), and tumour stage (TNM), and date of first ADT administration. Radiology reports were used to identify the presence of bone metastasis. This study was approved by an independent medical ethical committee (Academic Hospital Maastricht/University Maastricht METC 2019-1266). This research did not receive any specific grant from funding agencies in the public, commercial, or not-for-profit sectors.

### 2.3 Assessment of fracture risk

#### 2.3.1 Clinical risk factors and FRAX

The evaluation of clinical risk factors consisted of a detailed questionnaire, including questions regarding medical history and medication use, and fracture risk factors (e.g. previous fracture after the age of 50, number of falls in the past 12 months, parental hip fracture, current or past glucocorticoid use, current or past smoking and alcohol use, rheumatoid arthritis, and other diseases associated with secondary osteoporosis). At the time of the visit to the outpatient clinic, 10-year fracture risk for major osteoporotic fracture and hip fracture was assessed by the FRAX® tool using the online calculator for the Netherlands (https://www.sheffield.ac.uk/FRAX/tool.aspx?country=30). For the calculation of the FRAX score, BMD femoral neck values were included, while ADT (at initiation) was not considered as a cause of secondary osteoporosis, and prevalent VFs were not considered as a previous fracture [18].

#### 2.3.2 Bone mineral density measurement

BMD was measured at the lumbar spine (LS), total hip (TH) and femoral neck (FN) using dual energy X-ray absorptiometry (DXA) (Hologic QDR 4500, Hologic, Bedford, MA, USA). Osteoporosis was defined according to the WHO criteria as a T score ≤ -2.5 SD at either LS, TH or FN, osteopenia as a *T*-score between − 2.5 and − 1.0 SD, and normal bone density as a *T*-score ≥  − 1.0 SD. *T*-scores were calculated based on the female (Caucasian) reference database [19] according to the guideline of the International Society for Clinical Densitometry (ISCD) [20]. Additionally, a total body composition measurement was performed (see Section 2.2.3).

#### 2.3.3 Assessment of prevalent vertebral fractures

Lateral RX imaging of the thoracic and lumbar spine was performed in supine position. All radiographs were qualitatively analysed by radiologists to identify VFs and considered the potential differential diagnoses of a deformity, for instance metastasis or degeneration. Prevalent VFs were graded, according to method of Genant et al. [21] as mild 20–25 % (grade 1), moderate 25–40 % (grade 2) and severe ≥ 40 % (grade 3) height loss of the vertebral body. These were graded by a single radiologist and additionally by two experts in bone health who independently assessed all radiographs to identify VFs and discrepancies were resolved by consensus. For this study, a VF was diagnosed if there was a grade 2 or 3 height loss.

#### 2.3.4 Fracture risk-related comorbidities and medication

Known chronic comorbidities associated with an increased fracture risk according to the current national and international guidelines on osteoporosis and fall prevention (https://www.richtlijnendatabase.nl) were identified by reviewing medical history and medication use [22]. In addition, a blood sample was collected to detect new contributors to secondary osteoporosis and metabolic bone diseases [23]. Primary hyperparathyroidism was defined as hypercalcemia in the presence of inappropriately normal or elevated levels of parathyroid hormone and secondary hyperparathyroidism was defined as elevated plasma parathyroid hormone in the presence of 25(OH)D ≤ 50 nmol/l or CKD stage ≥ 3, or both [23].

Medication use was assessed and bone-related risk medication was identified. Bone related risk medication, defined by Vranken et al. [22] consisted of anticonvulsants, oral or inhaled glucocorticoids, H2-receptor inhibitors, proton pump inhibitors (PPI) and/or thiazolidinediones. Polypharmacy was defined as the use of at least 5 medications concurrently, in which ADT, dermatological preparations and medication that was not used chronically were not included [22], [24].

### 2.4 Nutrition, physical performance, and sarcopenia

#### 2.4.1 Nutritional status

The overall nutritional status was assessed by Mini Nutritional Assessment (MNA) [25]. The total score of the MNA distinguishes between adequate nutritional status (MNA ≥ 12), at risk of malnutrition (MNA between 11 and 7), and malnourished (MNA ≤ 8). The total daily dietary intake of calcium was calculated by the number of servings of dairy products consumed per day or week. A dietary calcium intake < 1000 mg per day was considered as inadequate. Body mass index was calculated from objectively measured weight and height (BMI, kg/m2).

#### 2.4.2 Physical activity, physical performance and muscle strength

Physical activity was defined according to the Dutch Physical Activity Norm (De Beweegnorm) (norm fit is ≥ 30 min of daily moderately intensive activities, semi fit is ≤ 30 min daily moderately intensive activities and non-fit is ≤ 30 min non-daily moderately intensive activities) [26]. All patients were asked for problems with keeping their balance, walking, or moving. Fear of falling was assessed by Dutch version of the Short Falls Efficacy Scale (Short FES-1), categorised into low concern (7–8 points), moderate concern (9–13 points) and high concern (14–28 points) [27]. Patients were asked to report their fitness by 0 to 10 Numeric Rating Scale (NRS). Measurements of physical performance and muscle strength were assessed in all patients who attended outpatient clinic and included handgrip-strength (HGS), timed up and go test (TUG) and the 30 s chair stand test (30CST). HGS was measured by using a Jamar handheld dynamometer. Patients were seated in a chair with the arm resting on armrest at 90 degrees with elbow in 90-degree flexion and the other arm in neutral position. Patients were asked to squeeze the dynamometer with maximal effort. The maximum HGS in kg was the highest score out of three attempts for their left and right hand and was used for analysis. A HGS<27 kg was defined as low [28]. For the TUG patients were observed and timed while they rise from a chair, walk around a pestle at 3  m, then walk back to the chair, and sit down. A score > 20 *sec*. is considered as at having an increased risk for a fall [28]. The 30SCST was performed by instructing patients to sit in a chair placed against the wall, with their hands on the opposite shoulder and their feet placed firmly on the floor, then on “go”, to stand in total upright position and then sit back down again. This was repeated for 30 s. The total number of complete chair stands (up and down is one stand) were counted. Cut-off point was ≤ 5 stands [28].

#### 2.4.3 Sarcopenia

Lean body mass was assessed using the body composition measurement by DXA (Hologic QDR 4500, Hologic, Bedford, MA, USA). The calculated appendicular lean mass (ALM) adjusted for height(ASM/height2) was used to assess muscle quantity. Sarcopenia was defined according to the definition of the EWGSOP2 [28]: I. Low muscle strength (criteria HGS < 27 kg or 30SCT ≤ 5 stands) II. Low muscle quantity or quality (criteria ASM/height < 7.0 kg/m2) and III. Low physical performance (criteria TUG > 20sec.). Probable sarcopenia is defined when criterion I is met. The diagnosis of sarcopenia is confirmed if criteria I and II are met; severe sarcopenia is diagnosed if criteria I, II, III are all met [28].

### 2.5 Statistical analysis

Data were analysed using descriptive statistics. All data were presented as means and standard deviations (SD) for continuous variables, number and percentage (%) for categorical variables. All analyses were performed using SPSS (version 24.0, IBM SPSS Statistics, USA).

## 3 Results

From January 2019 until December 2020, 125 consecutive patients with PCA were referred by urologist at the time of ADT initiation. Two patients cancelled the scheduled visit to the outpatient clinic because they were physically unable to attend. Out of 123 patients that were assessed according to the multidisciplinary care-pathway, eight patients were not eligible participation due to prior ADT use. In this study, 115 men with PCa were included with a mean age of 73.3 ± 7.6 years, who were using ADT for 56.5 (±34.0) days. Ninety men (78.3 %) had localised PCa and 25 (21.7 %) advanced PCa (Table 1). Polypharmacy was present in 44 (38.3 %) men. COPD and Diabetes Mellitus Type 2 were the most prevalent comorbidities (15.7 % and 14.8 %, respectively).

**Table 1:** Characteristics of men with PCa at initiation of ADT.

	Total (N=115)
Age (mean ± SD)	73.3 (±7.6)

BMI (mean ± SD)	27.8 (±4.1)

Localised, intermediate risk* (%)	18 (15.7%)
Localised, high risk* (%)	72 (62.6%)
Advanced disease (%)	25 (21.7%)

ADT indication (%)	
- Neo adjuvant RTX	18 (15.7%)
- Adjuvant RTX	72 (62.6%)
- Biochemical progression $	11 (9.6%)
- Recurrent PCa&	8 (7.0%)
- Progression (lymph-node) *	6 (5.2%)

**Medication**	

Current AOM	3 (2.6%)
Bone-related-risk medication^ (%)	
- 1 bone-related-risk medication	33 (28.7%)
- ≥2 bone-related-risk medication	9 (7.8%)

Polypharmacy ≥ 5 medications/day 3# (%)	44 (38.3%)

**Comorbidities**	

Rheumatoid Arthritis (%)	4 (3.5%)
Diabetes Type2 (%)	17 (14.8%)
COPD (%)	18 (15.7%)
CKD (stage 3,4) (%)	9 (7.8%)
Stroke (%)	12 (10.4%)

**Lifestyle**	

Smoking (%)	
- Never	29 (25.2%)
- Former	76 (66.1%)
- Current	10 (8.7%)
- Duration (years, mean, SD)	25.9 (±15.7)

Alcohol (%)	
- None	30 (26.1%)
- <20 units/week	76 (66.1%)
- >21 units/week	9 (7.8%)

Dietary calcium intake (SD,%)	
- Total mg/day (mean ± SD)	844 (±313)
- <1000mg/day (%)	84 (73%)

PCa = Prostate cancer ADT = Androgen Deprivation Therapy RTX = radiotherapy AOM = anti osteoporosis medication COPD = Chronic Obstructive Pulmonary Disorder CKD = Chronic Kidney Disease.

*Localised, intermediate risk = N0M0, T2, Gleason 7 or PSA 10–20, Localised, high risk = N0M0, T3, Gleason > 7 or PSA > 20).

^ Bone related risk medication = anticonvulsants, oral or inhaled glucocorticoids, H2-receptor inhibitors, proton pump inhibitors (PPI) and/or thiazolidinediones.

$ based on increased PSA.

& after radical prostatectomy.

* N1 = scan confirmed lymphatic metastases.

# ADT not included.

**Fig. 1:**
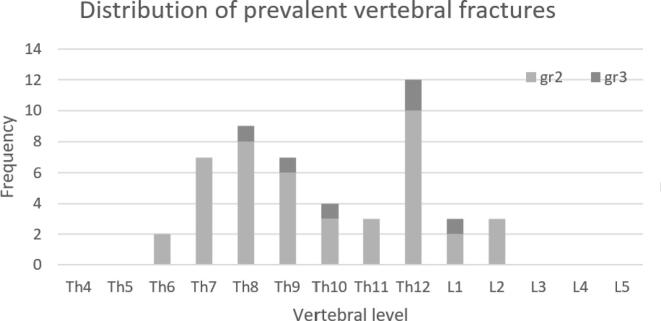
Distribution of prevalent vertebral fractures in men with PCa at initiation of ADT.

As shown in Table 2, osteoporosis was diagnosed in 5 men (4.3 %), osteopenia in 41 (35.7 %) and 69 (60.0 %) had a normal BMD. The calculated FRAX 10-year risk of a MOF and hip fracture was 4.4 %±2.8 % and 1.7 %±2.1 %, respectively. At least one fall incident in the past 12 months was reported by 24 (20.9 %) men. Based on the Dutch Osteoporosis Guideline AOM was initiated in 50 (43.5 %) patients. At least one moderate or severe VF was present in 37 men (32.2 %) with no difference between BMD categories (Table 3) and 39 men (33.9 %) had at least one grade 2 or 3 VF, osteoporosis or a combination. The VFs were most prevalent in the mid-thoracic (Th7-Th8) and thoracolumbar (Th12) area and the distribution levels are shown in Fig. 1. Further, at least one newly detected contributor to secondary osteoporosis and metabolic bone diseases excluding vitamin D deficiency was diagnosed in 12 (10.4 %) men (Table 4). Vitamin D insufficiency (31–50 nmol/l) was present in 28 (25.2 %) men and 5 (4.3 %) had a vitamin D deficiency (≤30 nmol/l). Secondary hyperparathyroidism (due to Vitamin D deficiency, CKD2 or both) was diagnosed in 11 (9.6 %) men.

**Table 2:** Prevalence of factors associated with fracture risk in men with PCa at initiation of ADT.

	Total (N = 115)
**DXA (Bone mineral density and ALM)**	

Osteoporosis^ (%)	5 (4.3 %)
Osteopenia (%)	41 (35.7 %)
Normal BMD (%)	69 (60.0 %)
BMD LS (mean ± SD)	1.12 (±0.21)
BMD FN (mean ± SD)	0.79 (±0.14)
BMD TH (mean ± SD)	0.96 (±0.15)
ALM/height^2^ < 7.0 (%)	16 (13.9 %)

**Vertebral fractures**	

At least one Grade 1,2,3 VF (%)	53 (46.1 %)
At least one Grade 2 or 3 VF (%)	37 (32.2 %)

**Fracture risk**	

Osteoporosis and/or at least one Grade 2 or 3 VF (%)	39 (33.9 %)
AOM intitiation$	50 (43.5 %)

**FRAX 10-year fracture risk**	

MOF % (mean ± SD)	4.4 (±2.8)
Hip fracture % (mean ± SD)	1.7 (±2.1)

**Risk factors**	

Previous fracture (at age ≥ 50y) (%)	7 (6.1 %)
Parental hip fracture (%)	9 (7.8 %)
One or more falls in the last 12 months (%)	24 (20.9 %)
Current smoking (%)	10 (8.7 %)
Current alcohol use > 3U / day (%)	9 (7.8 %)
BMI ≤ 20 kg/m^2^ (%)	3 (2.6 %)
Current use of glucocorticosteroids (%)	5 (4.3 %)

^Tscore ≤ -2.5 SD at either lumbar spine, total hip and femoral neck (female reference).

^#^Rheumatoid Arthritis, Diabetes type 2, COPD, Chronic Kidney Disease (stage 3,4), Stroke.

DXA = Dual X-ray absorptiometry ALM = Appendicular Lean Mass SD = standard deviation PCa = Prostate Cancer ADT = Androgen Deprivation Therapy MOF = major osteoporotic fracture, BMI = body mass index, LS = lumbar spine, TH = total hip, FN = femoral neck, VF = vertebral fracture AOM = anti-osteoporosis medication.

$ based on fracture risk criteria from the Dutch guideline on osteoporosis and fracture prevention (www.richtlijnendatabase.nl) with intervention thresholds: *T*-score ≤ -2.0 and/or ≥ 1 vertebral fracture grade 2,3.

**Table 3:** Prevalence of vertebral fractures in Bone mineral density categories.

	Osteoporosis N = 5	Osteopenia N = 41	Normal bone density N = 69
At least 1 gr 1,2,3 vertebral fracture*	2 (40 %)	24 (59 %)	27 (39 %)
At least 1 gr 2,3 vertebral fracture	1 (20 %)	14 (34.2 %)	23 (33.3 %)

*method of Genant et al. [21] as mild 20–25 % (grade 1), moderate 25–40 % (grade 2) and severe ≥ 40 % (grade 3).

**Table 4:** Newly detected vitamin D deficiency and comorbidities associated with fracture risk, in men with PCa at initiation of ADT.

Newly detected comorbidities	Total (N = 115)
Vitamin D insufficiency (25OHD 31–50 nmol/l) (%)	28 (25.2 %)
Vitamin D deficiency (25 OHD ≤ 30 nmol/l) (%)	5 (4.3 %)
Primary hyperthyroidism (%)	1 (0.9 %)
Primary hyperparathyroidism (%)	4 (3.5 %)
Secondary Hyperparathyroidism# (%)	11 (9.6 %)
CKD (stage 3,4) (%)	1 (0.9 %)
MGUS (%)	1 (0.9 %)
At least one new comorbidity (excluding 25OHD ≤ 30 nmol/l (%)	12 (10.4 %)

PCa = Prostate Cancer ADT = Androgen Deprivation Therapy CKD = Chronic Kidney Disease, MGUS = Monoclonal Gammopathy of Unknown Significance,

# due to Vit. D deficiency, CKD2 or Vit. D def. + CKD.

According to the MNA, one patient was malnourished and 73.6 % had an insufficient dietary calcium intake. One third of the men reached the physical activity norm. Self-reported difficulties in walking, moving or keeping balance were present in 27 (23.5 %) men and 14 (12.3 %) had high fear of falling. According to the EWGSOP2 11 men (10.1 %) had probable sarcopenia and only one (1) had confirmed sarcopenia (Table 5).

**Table 5:** Nutrition, Performance and Sarcopenia in men with PCa at initiation of ADT.

	Total = 115
Nutritional status according to MNA (%)	
- Normal nutritional status	102 (88.7 %)
- At risk of malnutrition	12 (10.4 %)
- Malnourished	1 (0.9 %)

Physical activity (NNGB) (%)	
- Non fit	62 (53.9 %)
- Semi norm fit	18 (15.7 %)
- Norm fit	35 (30.4 %)
- Fitness (NRS, mean)	7.1 (±1.3)

Difficulty moving/balance/walking (%)	27 (23.5 %)

Fear of falling (short FES)# (%)	
- High concern	14 (12.3 %)
- Low concern	100 (87.7 %)

Functional performance tests$ (%)	
- HGS < 27 kg	9 (8.3 %)
- CTS ≤ 5 times/30 *sec*.	4 (3.7 %)
- TUG > 20 *sec*	1 (0.9 %)

Sacropenia according to EGWSOP2 (%)	
- Probable sarcopenia	11 (10.1 %)
- Diagnosed Sarcopenia	1 (0.9 %)
- Severe sarcopenia	0

PCa = Prostate Cancer ADT = Androgen Deprivation Therapy BMI = Body Mass Index, MNA = Mini Nutritional Assessment, NNGB = Nederlandse Norm Gezond Bewegen, NRS = Numeric Rating Scale, FES = Falls Efficacy Scale HGS = Hand-Grip Strength, CTS = Chair Stand Test, TUG = Timed Up an Go, ASM = Appendicular Skeletal Muscle mass.

# 1 missing.

$ 7 missing due to COVID restrictions to perform physical consultations.

## 4 Discussion

In this cross-sectional study in men with PCa at the time of treatment initiation with ADT, one third had at least one moderate or severe VF, while only 4.3 % was diagnosed with osteoporosis, only one patient was diagnosed with sarcopenia and the 10-years fracture risk based on FRAX was low. In addition, 10 % of patients had at least one newly detected contributor to secondary osteoporosis and metabolic bone disease and thereby an increased fracture risk. In total, 39 men with PCa (33.9 %) had osteoporosis and / or at least one prevalent VF.

It is well known that patients with VFs have a high risk for future vertebral and non-vertebral fractures, even when these VFs are asymptomatic and not in the osteoporosis BMD category [29], [30]. However, evidence on fracture risk in men with PCa and prevalent VFs is disputable. Conversely, several guidelines include low BMD and/or prior fragility fractures (including VF) as a criterion for ‘high risk’ of non-metastatic fractures which are used to make clinical decisions regarding AOM initiation [12], [13], [14]. The prevalence of at least one moderate or severe prevalent VFs in our study (32.2 %) was higher as compared to the population based Rotterdam study [29], Tromsø study [30]and MrOs study [31], where at least one prevalent VF was reported in respectively 5–15 % of men. VFs in general are most prevalent at the mid-thoracic and thoracolumbar spine [30]. These parts of the spine endure the highest load during activities such as bending and lifting objects. In our study the VFs were most prevalent in the mid-thoracic (Th7-Th8) and thoracolumbar (Th12) area and the distribution of the VF levels in our study (Fig. 1) was similar to previous findings in the population of the Rotterdam Study [29]. However, the Rotterdam study included their participants between 1990 and 1993 and radiologic techniques have been improved since then. Conversely, the prevalence of VF is not extensively studied in PCa patients at the time of initiation of ADT treatment. In a previous study in men with locally advanced and/or metastatic and/or poorly differentiated PCa before starting ADT by Mistry at al. [32] a VF prevalence of 60 % was reported, but VFs were diagnosed and graded by the method of Eastell et al. Further, regarding the classification of fracture risk, Sullivan et al. [33] concluded that by adding a vertebral fracture assessment (VFA) to the systematic evaluation of fracture risk, 75 % of PCa patients had a high fracture risk, instead of 40 % based on BMD criteria alone. Greenspan et al [34] whom found a similar high VF prevalence of 38 % suggest that BMD alone would result in misclassification of high fracture risk in approximately 90 % of the cases. In contrast to our study, both studies [33], [34] included PCa patients with>6 months of ADT exposure and alterations in bone microarchitecture could already resulted in deterioration of bone quality. A recent real-life cross-sectional study by Mazziotti et al [35] in 98 men with PCa reported a prevalence of 45.9 % of VFs in men on ADT, but the prevalence of moderate/severe VFs in this study was 21.4 % and multiple in 14.3 %, which was lower than the prevalence of 32.2 % of moderate/severe VFs shown in our study. However, these findings cannot be compared in details since the men in the study by Mazziotti et al were younger (resp. 73.3 vs 61 years) and were already exposed to ADT. Although the design and methods of these studies and our study differ, it seems likely that the prevalence of VFs is substantially higher in men with PCa already at the time of initiation of ADT compared to the general population.

In our study, the majority of the moderate and severe VFs were found in the normal and osteopenia BMD categories, which is in line with the findings of Waterloo et al [30] who described that the risk of vertebral fractures was higher in men than in women in the highest BMD levels. Vertebral fragility fractures are often asymptomatic and overlooked but are known to decrease quality of life, and can also affect mortality [15]. These findings underline the importance of systematic imaging of the spine to detect prevalent VFs in PCa patients at initiation of ADT improving identification of true incident VFs during ADT.

The prevalence of osteoporosis in our study is lower as compared to previously reported prevalence rates of 6 % and 10 % in ADT naïve PCa patients [36], [37]. It should be noted that comparison with other PCa studies is challenging since most studies included patients with > 6 months of ADT exposure. Moreover, various reference databases (male or female) are used to calculate *T*-scores, which emphasises the need of uniformity regarding the use of the female reference database and should be included in PCa guidelines [19]. In case the female database to calculate *T*-scores is used, the prevalence of osteoporosis in our study is higher as compared to the 1.5 % in the population-based Tromsø study [38] and 2 % of MrOs study [31].

With respect to the clinical fracture risk factors (e.g. previous fracture, parental hip fracture, smoking, alcohol use, glucocorticoid use and rheumatoid arthritis), our results were comparable to the population based MrOs Sweden and MrOs US studies [39], [40]. Also, the 10-year FRAX probability for both a MOF and hip fracture (4.4 % and 1.7 %, respectively) was low in our study, and in line with the findings of Ojeda et al. [36] who reported a 10-year probability of 2.4 % for MOF, and 0.7 % for hip fractures at the time of PCa diagnosis and prior to ADT initiation. It should be noted that FRAX might underestimate fracture risk in men with PCa, especially since the number and severity of comorbidities, prevalent VFs and fall(risk) are not incorporated in the algorithm [41]. Although urology guidelines recommend the use of FRAX, they also address that calculation of FRAX fracture probability is not yet validated for PCa patients receiving ADT [14].

The prevalence of sarcopenia in our study is low, only one patient was diagnosed with sarcopenia, and 10 % was diagnosed with probable sarcopenia (based on low muscle strength). Muscle strength and physical performance (CST, TUG, HGS) and ALM were comparable to the population based MrOs studies [40]. The mean self-reported fitness VAS score in our study was 7.1 (0–10), implicating that at the time of ADT initiation, patients feel generally fit, confirming the results of the physical performance measurements. Moreover, a previous prospective study showed that the lean body mass declines significantly during ADT and continues to decline with long-term treatment [42] and the altered physical performance leads to an increased risk of falls [9]. Therefore a low prevalence of sarcopenia at initiation of ADT could lead to a delay in preventive strategies to mitigate or reverse the adverse effect of ADT.

In this cross-sectional study we presented real-life data which were systematically collected from patients receiving fracture risk evaluation immediately after initiation of ADT based on multidisciplinary care-pathway. We believe this study provides insight in the fracture risk and physical fitness of men at the time of ADT initiation, which is also the starting point of the clinical care-pathway. Some limitations of our study should be noted. First, this was a cross-sectional study and therefore we were not able to study the association of the various risk factors with incident fractures. Second, the decision to start ADT treatment depends on multiple factors such as underlying PCa risk profile, patient’s characteristics, physician’s and patient’s preferences, indicating selection of exposure to ADT and thereby introducing selection bias. Third, due to the nature of the descriptive design of our study the results have not been compared to a non-cancer, age-matched group. Fourth, our cohort was predominantly Caucasian, and the results may not be transferable to the general population. Finally, due to the COVID-19 pandemic lockdown we were not able to perform the physical performance tests in nine patients and diagnostic tests were postponed in 10 % of our patients.

Besides the limitations, at the time of ADT initiation, the prevalence of osteoporosis and sarcopenia is low and the FRAX 10-year fracture risk score, and physical performance test are mostly normal. However, a third of men with PCa already had at least one moderate or severe VFs, mostly with a BMD in the normal or osteopenic range. Consequently, at the time of initiation of ADT, men already have prevalent VFs even before the detrimental effects of ADT on bone will increase fracture risk. When fracture risk assessment is based on BMD and/or FRAX scores, without vertebral fracture assessment, there is a risk of under-identification of men who could experience benefits of AOM treatment but also to classify any subsequent fractures reliably as incident rather than prior events. We therefore would like to emphasize the importance of a vertebral fracture assessment next to a BMD measurement and FRAX risk calculation in men with PCa at initiation of ADT.

## 5 Conclusion

Although the prevalence of osteoporosis, sarcopenia and calculated 10-years fracture risk is low, there is a high prevalence of vertebral fractures in a third of the men with PCa at the time of ADT initiation. Besides a BMD measurement and / or FRAX® risk calculation, systematic vertebral fracture risk assessment should be considered in all men with PCa at initiation of ADT to provide a reliable baseline classification of VFs improving identification of true incident VFs arising during ADT.

## Declaration of Competing Interest

The authors declare the following financial interests/personal relationships which may be considered as potential competing interests: Marsha van Oostwaard, Dr. Yes van de Wouw, Professor dr. Maryska Janssen-Heijnen, Dr. Marc de Jong and Dr. Caroline Wyers have no conflicts of interest. Professor dr. Joop van den Bergh reports consulting fees and payment or honoraria for lectures, presentations, speakers bureaus, manuscript writing or educational events from Amgen institution and UCB institution outside the submitted work.
